# Platelet-rich plasma treatment improves postoperative recovery in patients with pilonidal sinus disease: a randomized controlled clinical trial

**DOI:** 10.1186/s12893-021-01370-5

**Published:** 2021-10-21

**Authors:** Can Yahya Boztug, Tulay Karaagac Akyol, Cigdem Benlice, Mehmet Ali Koc, Beyza Doganay Erdogan, Osman Ilhami Ozcebe, Mehmet Ayhan Kuzu, Cihangir Akyol

**Affiliations:** 1 Department of Surgery, Ankara University Faculty of Medicine, Ankara, Turkey; 2 Department of Hematology, Hacettepe University Faculty of Medicine, Ankara, Turkey; 3 Department of Biostatistics, Ankara University Faculty of Medicine, Ankara, Turkey

**Keywords:** PRP, Pilonidal sinus, Quality of life, Pain

## Abstract

**Background:**

Pilonidal sinus is a common health problem. The current study aimed to compare the impact of autologous platelet-rich plasma (PRP) with that of minimally invasive techniques in terms of pain reduction, return to daily activities, quality of life, and duration of wound healing after open excision and secondary closure.

**Methods:**

Patients who were over 18 years old and had chronic PS disease between March 2018 and January 2019 were enrolled and randomly divided into three groups. Open surgery and moist dressings were applied to patients in group A. Open surgery followed by PRP application was performed on patients in group B. Group C underwent curettage of the sinus cavity followed by application of PRP. In this prospective randomized controlled study, patients completed questionnaires (including the Nottingham Health Profile (NHP), Short Form-36 (SF-36) and clinical information) before and after surgery. Demographics, preoperative characteristics, healing parameters, and quality-of-life scores were evaluated and calculated before and after surgery.

**Results and conclusion:**

The cavity volume and wound-healing time were compared among the groups on postoperative days 0, 2, 3, 4, and 21. Each patient was followed up throughout the process of wound healing, and follow-up was continued afterward to monitor the patients for recurrence. Due to the nature of the treatment that group C received, this group achieved shorter healing times and smaller cavity volume than the other groups. In contrast, the recovery time per unit of cavity volume was significantly faster in group B than in the other groups. Overall postoperative pain scores were significantly lower for both PRP groups (open surgery, group B; minimally invasive surgery, group C) than for group A (p < 0.001) and showed different time courses among the groups. In the treatment of PS disease, PRP application improves postoperative recovery in that it speeds patients’ return to daily activities, reduces their pain scores and increases their quality of life.

*Trial registration* The current study is registered on the public website ClinicalTrials.gov (ClinicalTrials.gov identifier number: NCT04697082; date: 05/01/2021).

**Supplementary Information:**

The online version contains supplementary material available at 10.1186/s12893-021-01370-5.

## Introduction

Pilonidal sinus (PS) disease is a health problem that has been attacked using various treatment modalities since it was first described by Herbert Mayo in 1833. PS is most commonly diagnosed at 30 years of age, and 70–80% of patients are male [1].

Common morbidities after the surgical treatment of PS are pain, loss of productive work hours and wound infections owing to long healing times [2]. Therefore, the main goals of treatment are to accelerate healing by decreasing pain and to reduce the loss of productive work hours. Local administration of platelet-rich plasma (PRP), which contains growth factors (GFs), is a new method that has been reported to accelerate the healing process by 30–40% [3]. In this study, we aimed to investigate the effect of PRP on patients’ pain scores, wound healing and quality of life in the process of treatment for PS disease.

## Methods

### Trial design

We designed the trial as a prospective, randomized controlled study. This study was conducted in accordance with the Declaration of Helsinki and was started after approval from the Ethics Committee of Ankara University Medicine Faculty in Ankara (Approval Number: 03-162-18). The current study is also registered on the public website ClinicalTrials.gov (ClinicalTrials.gov identifier number: NCT04697082; date: 05/01/2021). Informed consent was obtained from all the participants.

### Participants and eligibility criteria

Patients who were over 18 years old and had chronic PS disease were included in the study. We excluded patients if they met any of the following criteria: acute abscess, anaemia, use of immunosuppressive drugs, haematological malignancy, bleeding disorders or recurrent PS disease.

### Randomization

The patients were randomly divided into three groups. Simple randomization was performed using computer-generated random numbers in Microsoft Excel [4]. The interventions for each group are described below:

Group A: Open surgery and moist dressings were applied to these patients.

Group B: These patients underwent open surgery followed by PRP application. At the end of the procedure, the PRP-filled cavity was covered with PRP-impregnated gauze.

**Fig. 1:**
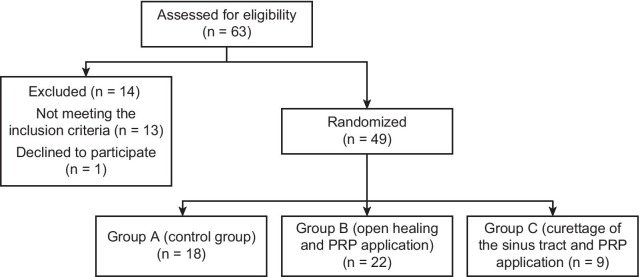
CONSORT diagram

Group C: This group underwent curettage of the sinus cavity followed by PRP application. At the end of the procedure, the PRP-filled sinus tract was covered with PRP-impregnated gauze (Fig. 1).

### Medical history and demographics

After enrolling the patients, we recorded their demographic data, including gender, age, education, comorbid diseases and drug use. Then, the Short Form-36 (SF-36) and Nottingham Health Profile (NHP) quality-of-life score questionnaires were administered before surgery. All patients were asked to remove the hair from the sacrococcygeal region using depilatory cream.

### Open surgical procedure

**Fig. 2:**
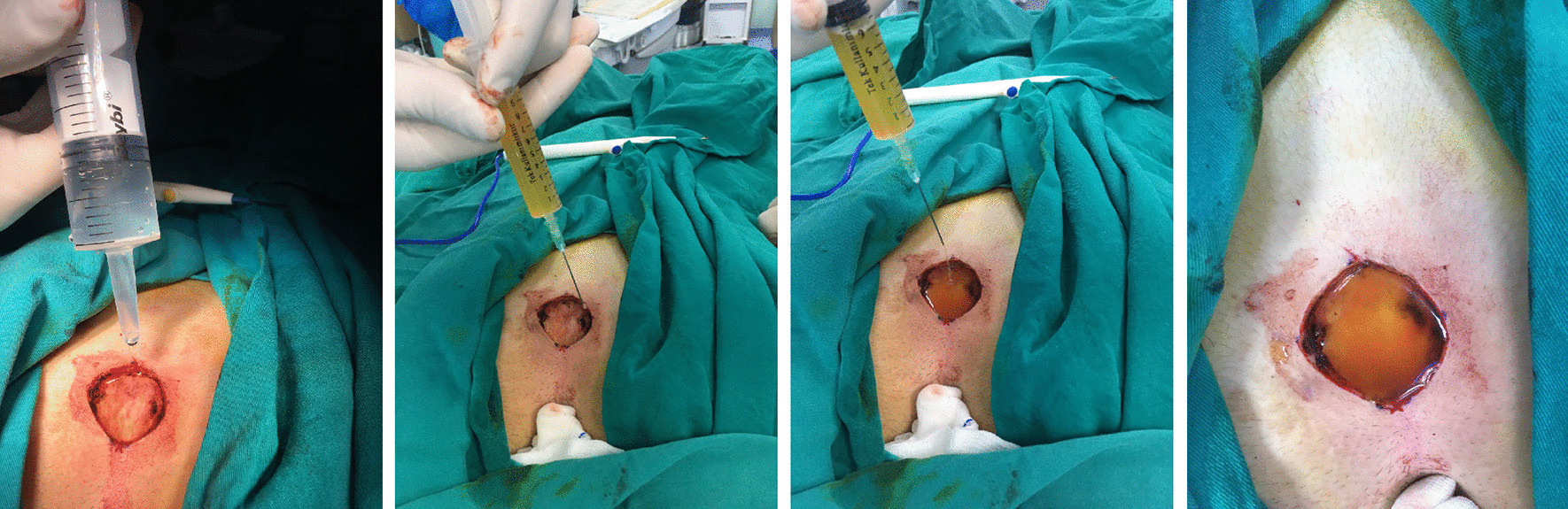
Measurement of cavity volume and local PRP administration

Each patient entered the operating room, and after the administration of general anaesthesia, the patient was placed in the prone position. Then, the buttocks were pulled laterally using adhesive bands to enable the removal of the diseased area. The sacrococcygeal region was cleaned and disinfected with 10% povidone-iodine. After the surrounding area was covered, the sinus tract was examined by using a thin steel cane. The length and width of the cavity were noted. Then, the sinus tract was removed, and the cavity volume was measured (Fig. 2).

### Minimally invasive surgery

**Fig. 3:**
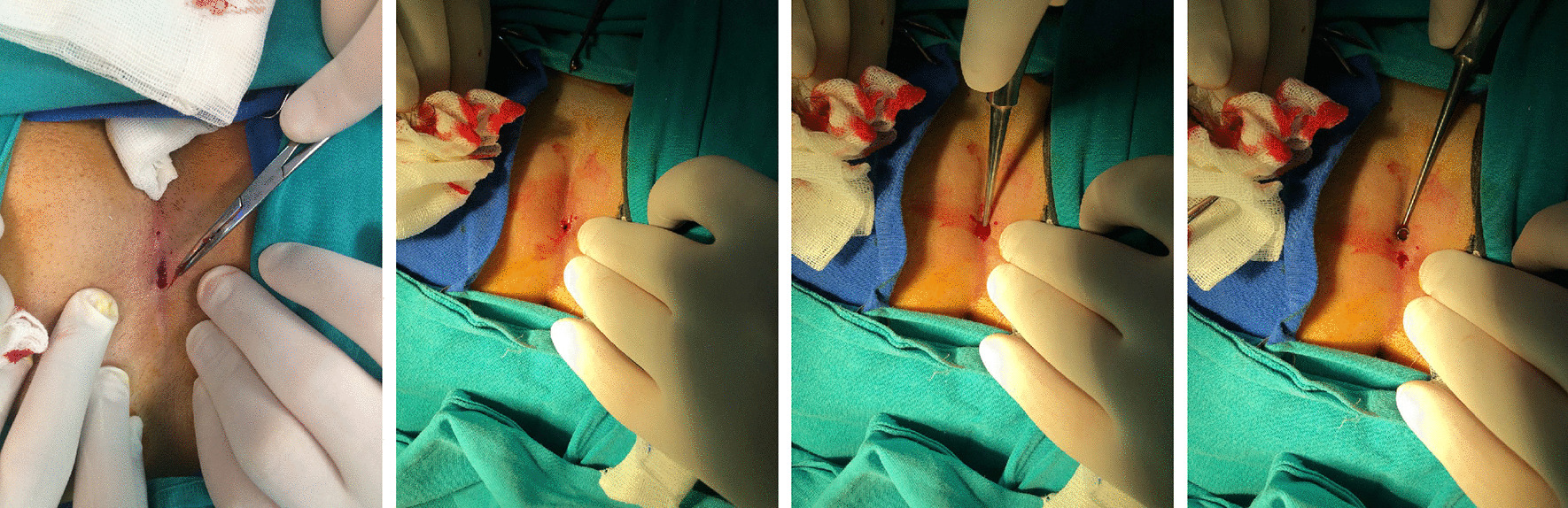
Minimally invasive surgery

Minimally invasive surgery was performed under local anaesthesia in the operating room. The patient was prepared as for the open technique. The largest pit was excised, and all hairs in the cavity were removed with forceps (Fig. 3). Then, the whole tract was curetted meticulously and irrigated with saline solution. After haemostasis, the cavity volume was measured, and PRP was applied to the cavity.

For group C, the cavity volume was low due to the nature of the operation. Therefore, we used a new parameter to standardize the cavity volume and wound-healing time. This new parameter was the recovery time per cavity (day/cc). A similar parameter was previously used in a study by Spyridakis et al. [5]. This ratio is the most objective parameter for comparison.

#### Measurement of cavity volume

The depth of the cavity was noted. Then, 50 cc of 0.9% saline solution was loaded into a syringe without inserting the needle into the tissue and added to the cavity until it filled the whole space, allowing the cavity volume to be measured accurately (Fig. 2). We didn’t create any microtrauma during the measurement. The measurement was performed by subtracting the remaining volume of saline solution in the syringe from the total syringe volume, which was 50 cc.

### PRP administration

After the cavity volume was measured with 0.9% saline solution, PRP was administered by filling the whole space from the lateral side of the cavity without creating any microtrauma. After the first application of PRP, the second dose was applied at 48 h. Until that time, the dressing was not removed. Additional PRP was applied on the 3rd, 4th and 5th days postoperatively.

### Preparation of PRP

For clinical applications, good manufacturing practice–compliant human platelet concentrates can be created by the standard method for PRP production. Erythrocyte concentrate and platelet-containing plasma are separated by the first centrifugation, and platelet-poor plasma (PPP) and PRP/platelet concentrate are created by centrifuging the platelet-containing plasma a second time [6]. Platelets contain coagulation factors, growth factors (PDGF, TGF-β1, IGF-1, VEGF, FGF-2) and interleukins (IL-1, IL-4, IL-6, IL-10, IL-13) [7].

**Fig. 4:**
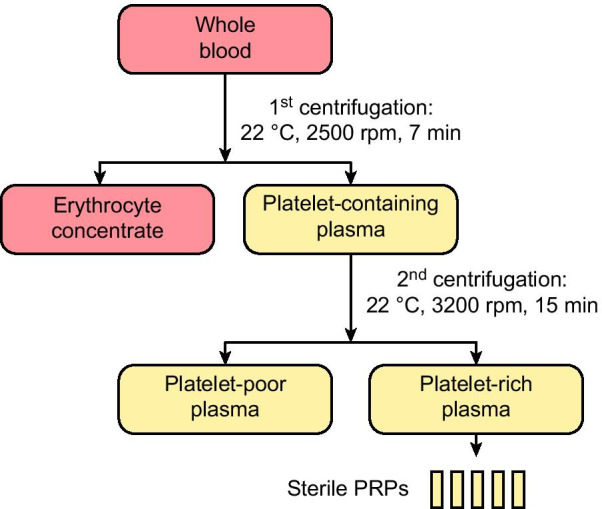
PRP preparation steps

We prepared the platelet concentrate by the PRP production method in the Hacettepe University Blood Center Laboratory, applying all standardized processes for healthy blood donors. In accordance with the “National Blood and Blood Components Preparation Use and Quality Assurance Guidelines”, the requirements for PRP include a volume of at least 40 mL, a minimum platelet count of 60 × 10^9^/unit [8], and a maximum leucocyte count of 0.2 × 10^9^/unit [9]. After approval, 450 cc of blood was drawn from the patient by phlebotomy on the morning of surgery. A two-stage centrifugation method was used to separate the whole blood into its components. In the first stage, plasma was separated from erythrocyte concentrate by low-speed centrifugation in a temperature-controlled Heraeus Cryofuge 6000i (22 °C, 2500 rpm, 7 min). After a 1-h waiting period, the second stage was initiated, in which the PRP/platelet concentrate was separated from the PPP by high-speed centrifugation (22 °C, 3200 rpm, 15 min). The PRP/platelet concentrate obtained after the second centrifugation step was shaken on an agitator for 1 h and divided into 5 equal parts with a sterile connection device (Fig. 4). We preserved the PRP at 25 °C on agitators. Before each dressing was placed, we applied a PRP aliquot to the wound with a syringe.

### Postoperative care

All patients were discharged on the 1st postoperative day. Patients in all groups were advised not to uncover the wound for 2 days. If the patients experienced pain, they were advised to take the prescribed pain reliever no more than 2 times a day.

#### Healing process

At every postoperative visit, the cavity volume was measured. Wound healing time was standardized as the recovery time per unit of cavity volume (days/cc), an index that was previously used in the research of Spyridakis et al. [5].

### Questionnaires

The SF-36 and NHP questionnaires were applied to evaluate the patients’ quality of life before and 3 weeks after surgery. We assessed the patients’ general health, limitations, activities, physical health problems, emotional health problems, social engagement, pain, energy and emotions with the SF-36 questionnaire. Additionally, we evaluated the patients’ pain, energy levels, emotional reactions, sleep, social isolation, and physical abilities of patients with the NHP questionnaire.

We used a visual analogue scale (VAS) to evaluate pain scores. This scale ranged from 0 to 10, with higher scores indicating a stronger sensation of pain. The VAS scale was applied five times after surgery, on postoperative days 1, 2, 4, 5 and 21. Patients were routinely prescribed dexketoprofen trometamol and advised to take it no more than 2 times a day. The number of tablets each patient took after the operation was recorded. The time interval until the patients became able to walk without pain (labelled herein as the “pain-free walking time”) was recorded, as was the interval until the patients returned to their preoperative daily activities without any strain.

### Statistical analysis

Nonparametric tests were used for hypothesis testing due to the small sample size and skewed data distribution. Numerical variables are summarized as the mean ± standard deviation or as the median (minimum–maximum). Frequencies and percentages were used to summarize categorical variables. Three groups were compared with regard to demographic and clinical characteristics of patients using the Kruskal–Wallis test, Pearson’s Chi-square test or Fisher’s exact test. Dunn’s post hoc test was performed to determine which groups were different from others after a Kruskal–Wallis test. Since pre- and postoperative observations of the same patient are dependent, we used a robust rank-based nonparametric method proposed by Bruner and Puri [10] for the analysis of longitudinal data in factorial contexts. With this method, relative treatment effects (RTEs) were given as descriptive point estimators. The 95% confidence intervals (CIs) of RTEs were also used to make post hoc inferences. If the related 95% CIs did not overlap, we concluded that there was a significant difference between the time points or groups compared. The RTE is the probability that a randomly chosen observation from the time point and/or group under consideration has a larger value than a randomly chosen observation from the whole dataset regardless of the time point and/or group under consideration. The F1_LD_F1 design was used to analyse the repeated measurements taken from patients in the three groups. We tested three null hypotheses: no main effect of time, no main effect of group and no interaction effect of time and group. If the null hypothesis of no effect is true, every group and time point should have an RTE of 0.50. When the interaction effect is significant, it means that the trend in the observations over time is different between groups. When the main effect of time is significant, the observations differ across time points without regard for the group labels. When the main effect of group is significant, the observations differ across groups without regard for the time variable. All analyses were performed in R 3.4.4 (R Development Team), and the nparLD package was used for nonparametric repeated F1_LD_F1 design. A p value of < 0.05 was considered to indicate a statistically significant difference [11, 12].

#### Post hoc power analysis

Power analysis was performed according to the primary aim of the study, which was to compare surgery groups (A, B, and C) in terms of healing parameters (Table 2). The power of the test was calculated as 98% for an effect size of f = 0.64 (which is considered a large value and was calculated using the means in Table 2) and a Type I error rate of 0.05, with the number of groups set to 3 and the sample sizes in the groups specified as 21, 18, and 9. Power analysis was performed using the G*Power v.3.1 program.

## Results

Between March 2018 and January 2019, 49 of 63 recruited patients were included in the study, and 14 patients were excluded. There were 18 patients in group A, 22 patients in group B and 9 patients in group C. One patient in group C was excluded because this participant was lost to follow-up. There was no significant difference in the demographic characteristics of the patients by group (Table 1).

**Table 1:** Demographic characteristics of the patients

	Group A	Group B	Group C	p
Age (years)	26.7 ± 5.5 27 (18–39)	24.7 ± 5.5 23.5 (18–37)	26.1 ± 10 23 (18–49)	0.400
Gender				0.808
Female	4 (22.2%)	5 (22.7%)	1 (11.1%)	
Male	14 (77.8%)	17 (77.3%)	8 (88.9%)	
Education level				0.004
Primary	1 (5.6%)	0	1 (11.1%)	
High school	7 (38.9%)	8 (36.4%)	5 (55.6%)	
College	10 (55.5%)	14 (63.6%)	3 (33.3%)	
Chronic disease	2 (11.1%)	4 (18.2%)	4 (44.4%)	0.153
Chronic drug usage	1 (5.6%)	2 (9.1%)	3 (33.3%)	0.136
Family history of PS	5 (27.8%)	6 (27.3%)	1 (11.1%)	0.750

*PS* pilonidal sinus

**Fig. 5:**
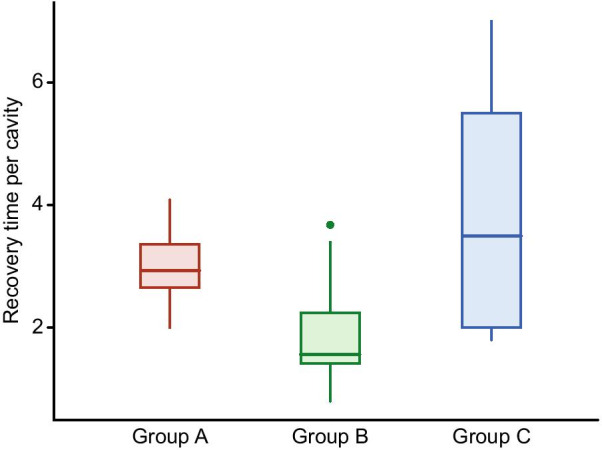
Recovery time per unit of cavity volume (day/cc)

The healing parameters of the groups are shown in Table 2. While the cavity volume did not differ between groups A and B (18.8 ± 8.2 cc vs. 22.6 ± 11.5 cc; p = 0.393), the recovery time per unit of cavity volume was significantly faster in group B than in group A (p < 0.001) (Fig. 5). However, there was no significant difference between group A and group B regarding wound-healing time (p = 0.092). Despite the minimally invasive technique and reduced cavity volume in group C, the recovery time per unit volume was slower in that group than in the other two (2.9 vs. 1.8 vs. 3.8 days/cc for groups A, B, and C, respectively; p < 0.001).

**Table 2:** Healing parameters of the groups

	Group A	Group B	Group C	p
Total number of pits	3.56 ± 1.5 3.5 (1–8)	3.6 ± 1.5 3 (1–8)	2.0 ± 0.7 2 (1–3)	**0.004**^**β**,**γ**^
Cavity volume (cc)	18.8 ± 8.2 18 (8–39)	22.6 ± 11.5 21 (5–45)	4.0 ± 1.4 4 (2–7)	**< 0.001**^**β**,**γ**^
Wound healing time (day)	54.4 ± 24.3 48 (23–114)	37.1 ± 16.6 34 (16–81)	13.9 ± 6.6 11 (8–28)	**< 0.001**^**β**,**γ**^
Recovery time per unit of cavity volume (days/cc)	2.9 ± 0.6 2.9 (2–4)	1.8 ± 0.7 1.5 (0.8–3.6)	3.8 ± 2.1 3.5 (1.8–7)	**< 0.001**^**α**,**γ**^

^α^There was a significant difference between Group A and Group B after post hoc analysis

^β^There was a significant difference between Group A and Group C after post hoc analysis

^γ^There was a significant difference between Group B and Group C after post hoc analysis

In the postoperative period, the need for painkillers was significantly lower in group B and group C than in group A (p = 0.003 and p < 0.001, respectively). Additionally, the time required to return to normal activity was significantly shorter in both PRP groups (B and C) than in group A (p = 0.003 and p < 0.001, respectively) (Table 3).

**Table 3:** Comparison of the patients with respect to the use of painkillers, pain-free walking time, and time taken to return to daily activities

	Group A	Group B	Group C	p
Quantity of painkillers needed	9.5 ± 4.3 10 (0–14)	3.4 ± 2.5 4 (0–10)	0.1 ± 0.3 0 (0–1)	**< 0.001**^**α**,**β**,**γ**^
Pain-free walking time	6.4 ± 2.3 6 (3–12)	5.3 ± 2.3 6 (1–12)	2.7 ± 0.7 3 (2–4)	**< 0.001**^**β**,**γ**^
Time taken to return to daily activities	16.3 ± 5.6 15.5 (7–30)	9.0 ± 4.5 8 (3–20)	3.0 ± 1.1 3 (2–5)	**< 0.001**^**α**,**β**,**γ**^

^α^There was a significant difference between Group A and Group B after post hoc analysis

^β^There was a significant difference between Group A and Group C after post hoc analysis

^γ^There was a significant difference between Group B and Group C after post hoc analysis

**Fig. 6:**
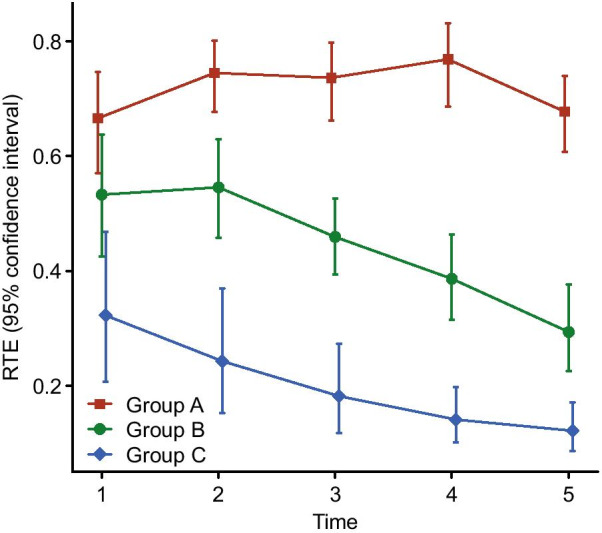
Graph showing VAS scores by RTE. If the relevant 95% CIs did not overlap, we concluded that there was a significant difference between the time points or groups compared

When VAS scores were compared, it was found that there was no significant difference in these scores on the 1st day. When the difference between groups was investigated by measurement time, it was determined by testing whether the RTEs intersected the 95% CIs (Fig. 6). From visit 2 to visit 5, the VAS scores group B and group C were significantly lower than those of group A. There was no significant group difference in preoperative–postoperative changes in SF-36 or NHP quality-of-life scores. However, when the pain and general health perception parameters of the SF-36 were considered in isolation, these parameters were observed to be significantly improved in group B from the preoperative to the postoperative period.

All patients were followed up for 6 months. There were no recurrences of disease in any of the groups. Four patients in group C had abscesses after the fifth PRP dose was applied (Additional file 1).

## Discussion

Both PS disease and its treatment process can cause some complications. For example, pain during dressing changes, bad odour during the healing process and fear of recurrence of the disease cause serious concern in patients [13, 14]. Therefore, the optimal treatment modality for patients should be painless, allowing them to return to their daily activities quickly and with a minimal recurrence rate [15]. However, surgeons are still working to determine the most appropriate method, evaluating minimally invasive methods and various other treatments [16]. Recently, there has been an emphasis on imaging and physical examination studies aiming to provide objective criteria in the diagnosis, treatment and follow-up of PS [17].

The present study compared the effectiveness PRP with that of conventional moist dressings and included a third group (the minimally invasive group) that was not found in previous studies on PRP application in PS disease. With or without PRP, we favour the open healing process because of the low recurrence rate [13]. The aim of our study was to evaluate the effect of PRP on the wound-healing process in the PS cavity after surgery. An accelerated recovery time reduces the risk of infection in the surgical region and would reduce the loss of productive work hours [5]. Additionally, we aimed to assess the level of pain following each procedure.

The primary end point of this study was the completion of wound healing, which was significantly faster in group B and group C than in group A. However, this parameter is not objective enough because there was a considerable difference in cavity volume between groups. When we compared the cavity volumes among all three groups, there was no significant difference between group A and group B, whereas in group C, the cavity volume was found to be significantly lower due to the nature of the surgical technique. Therefore, we needed an objective parameter to compare the complete wound-healing times of group B and group C. For this purpose, we used the recovery time per unit of cavity volume. This index represents the number of days required for each 1 cc of cavity volume to be filled. We found that the recovery time per unit of cavity volume was 2.9 days/cc in group A, 1.8 days/cc in group B and 3.8 days/cc in group C. We believe that the reason group C needed more recovery time per unit volume despite the small absolute volume was that the PRP was trapped in the cavity and was not dispersed and drained sufficiently. We believe this is the reason why 4 patients in this group had abscesses in the 2nd week of follow-up. Since there was an almost 50% incidence of abscess development, patient admission to group C was stopped, and the study was continued with the other two groups (group A and group B).

PRP treatment gained popularity in regenerative medicine and other fields with the publication of early outcomes of its use in maxillofacial surgery and cardiac surgery in 1980–1990 [18, 19]. PRP has been shown to significantly accelerate the recovery process, especially in tissues with weak blood flow and slow cell cycles, such as tendons, ligaments and cardiac tissues [20, 21]. Sun et al. evaluated the therapeutic effects of autologous PRP on deep partial-thickness burns in Bama pigs. Their data showed that the time to wound re-epithelialization was shortened in the PRP group [22]. In another study, PRP was combined with chitosan and silk fibroin to prevent the activity of proteases in the wound microenvironment, and this preparation was applied to diabetic ulcers. It was determined that repair cells proliferated rapidly under similar conditions in vitro, and angiogenesis and nerve repair were accelerated in vivo [23]. There are also studies investigating the preventive effect of PRP on anastomotic leaks based on its positive effect on wound healing. In such a study on rats, the effect on anastomotic leakage after intraperitoneal chemotherapy was investigated, and anastomotic burst pressure was found to be significantly different in the PRP group [24].

PRP exerts an effect on the wound-healing process locally by delivering growth factors and cytokines to the wound area; these are the main elements of healing in the stages of inflammation, proliferation and remodelling [25]. Growth factors are proteins that serve as signalling molecules for cells. The main source of growth factors in PRP is platelets. Platelet-derived growth factor (PDGF) stimulates cell replication and promotes angiogenesis, epithelization, and granulation tissue formation. Vascular endothelial growth factor (VEGF) promotes angiogenesis. Epidermal growth factor (EGF) promotes cell differentiation and stimulates re-epithelization, angiogenesis and collagenase activity. Fibroblast growth factor (FGF) promotes the proliferation of endothelial cells and fibroblasts and stimulates angiogenesis. Transforming growth factor beta (TGF-β) 2 and 3 promote the formation of the extracellular matrix [26–30].

The comparison of VAS scores revealed different trends in the three groups over time. The reduction in pain in group B and group C from the 2nd day onward suggests that the analgesic effect of PRP started after the 24th hour postoperatively. The low VAS scores beginning on the second day reduced the need for painkillers. There have been only a few studies on the use of PRP in PS; these studies evaluated the effect of PRP application on the wound-healing process, pain and the ability to return to daily activities after open surgery [5, 31]. In both studies, wound-healing time, pain scores and time to return to daily activities were more favourable in the PRP-treated group than in the non-PRP-treated control group. These results are in line with the findings in our study, showing a rapid return to daily activity, low pain scores and rapid wound healing. Furthermore, the effectiveness of PRP as an analgesic has been demonstrated in painful conditions such as osteoarthritis and trochanteric pain syndrome [32, 33].

The SF-36 and NHP questionnaires were also evaluated based on 95% CIs of the RTEs. When the questionnaire results were evaluated globally, no significant difference was detected between the preoperative and postoperative periods. However, when we considered the subscales of the SF-36 separately, we found confirmation of the effect of PRP on pain. SF-36 pain scores were significantly improved in group B. Additionally, in this group, the general health perception parameter of the SF-36 was significantly improved. While differences were found in these two parameters, no significant differences were identified in the other six parameters of psychological distress assessed by the SF-36. In the study of Spyridakis et al., when global SF-36 scores were evaluated, it was found that the PRP group had a lower level of psychological distress than the control group [5].

## Limitations

This study has several potential limitations, which need to be addressed in future studies. We are aware that the present study had a smaller sample than other, similar studies. In addition, group C had fewer patients than either of the other groups because we stopped allocating patients to group C due to the high rate of postoperative abscess formation. The absence of a minimally invasive non-PRP-treated control for group C is another limitation. Cost-effectiveness and cost–benefit analyses were not performed because the main goal of the study was not to evaluate these parameters. Another limitation is that the patient follow-up times varied between 6 and 18 months.

## Conclusion

In the treatment of PS disease, PRP application improves postoperative recovery in that it speeds patients’ return to daily activities, reduces their pain scores and increases their quality of life.

The use of PRP for wound care during postoperative healing by secondary intention should be considered in the surgeon’s armamentarium for the management of PS disease.

## Supplementary Information

**Additional file 1.** : Consent Form and Follow-up Form.

## Data Availability

The datasets used and analysed during the current study are available from the corresponding author upon reasonable request.

## Abbreviations

PRP: Platelet-rich plasma
PS: Pilonidal sinus
GFs: Growth factors
SF-36: Short Form-36
NHP: Nottingham Health Profile
VAS: Visual Analogue Scale
PPP: Platelet-poor plasma
PDGF: Platelet-derived growth factor
EGF: Epidermal growth factor
IGF-1: Insulin-like growth factor-1
VEGF: Vascular endothelial growth factor
FGF-2: Fibroblast growth factor-2
TGF-β: Transforming growth factor beta
IL-1: Interleukin-1
IL-4: Interleukin-4
IL-6: Interleukin-6
IL-10: Interleukin-10
IL-13: Interleukin-13
RTEs: Relative treatment effects
CIs: Confidence intervals
